# Acetylene Semi‐Hydrogenation by Photocatalysis

**DOI:** 10.1002/anie.5613575

**Published:** 2026-08-16

**Authors:** Anna Fortunato, Brando Adranno, Sharon Silloni, Marianna Barbieri, Claudia Masia, Francesca Arcudi, Luka Đorđević

**Affiliations:** ^1^ Department of Chemical Sciences University of Padova Padova Italy

**Keywords:** acetylene, ethylene, heterogeneous catalysts, homogeneous catalysts, photocatalysis

## Abstract

Acetylene to ethylene semi‐hydrogenation is a cornerstone process in the production of polymer‐grade feedstocks, yet it remains dominated by energy‐intensive noble metal‐based thermocatalysis. Photocatalysis is now emerging as a compelling alternative, using light to generate reactive intermediates under mild conditions and replacing hydrogen gas with safer proton sources such as water or alcohols. Although still in its infancy, this field is rapidly evolving, with a growing number of studies already achieving remarkable performances in both selectivity and efficiency. This Minireview provides an overview of photocatalytic acetylene semi‐hydrogenation, framing recent advances within a broader industrial context. Emphasis is placed on mechanistic understanding, comparing π‐complex, hydrogen atom transfer (HAT), and concerted proton‐coupled electron transfer (CPET) pathways alongside the experimental tools used to distinguish them. Recent developments in both homogeneous and heterogeneous catalytic systems are discussed and key challenges related to efficiency, scalability, and techno‐economic viability are presented, outlining design principles for next‐generation, earth‐abundant photocatalysts, and identifying opportunities for the transition from impurity removal to direct acetylene valorization.

## Graphical abstract

Photocatalytic acetylene semi‐hydrogenation is an emerging light‐driven route to polymer‐grade ethylene, offering a mild alternative to energy‐intensive thermocatalysis. By replacing H_2_ co‐feed with water or other proton sources, these systems can achieve high selectivity under ambient conditions. This Minireview summarizes the mechanistic principles, benchmarks current catalytic platforms, and outlines key design principles and remaining challenges for scalable and sustainable implementation.

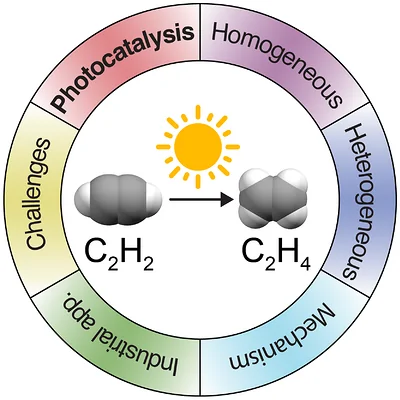

## 1 Introduction

Ethylene (C_2_H_4_) is one of the most important building blocks in the chemical industry, with a global annual output approaching 200 million tons [1], underpinning the production of approximately 60% of all plastics [2]. At present, its large‐scale manufacturing is dominated by high‐temperature steam cracking of hydrocarbon‐based feedstocks, a process that is both energy‐intensive and associated with substantial carbon emissions [3, 4, 5]. Beyond its environmental footprint, ethylene obtained from steam‐cracking inevitably contains impurities such as acetylene (C_2_H_2_, 0.5–2 vol%) [6]. This impurity must be removed to meet polymer‐grade specifications (<5 ppm), since acetylene acts as a poison for Ziegler–Natta polymerization catalysts [7, 8].

Traditional industrial purification of crude ethylene streams is achieved by thermocatalytic hydrogenation of acetylene to ethylene. Although this technology is well established [9, 10, 11], it typically requires elevated temperature and pressure, a supplemental hydrogen (H_2_) feed, and Pd‐based noble‐metal catalysts, which can promote over‐hydrogenation of ethylene to ethane. Since ethane also interferes with downstream polymerization, it must be removed from the ethylene stream before plastics production, adding costly, and energy‐intensive separation steps, such as cryogenic distillation [12]. These limitations reduce the sustainability and energy efficiency of the process, motivating the development of alternative strategies that operate under mild conditions, are powered by sustainable energy inputs, use green proton sources, and rely on inexpensive catalytic materials.

Over the years, both separation‐based and catalytic strategies have been explored to reduce the C_2_H_2_ content in C_2_H_4_ co‐feeds to below 5 ppm. Physisorption‐based approaches can efficiently remove acetylene, but they do so by separating and discarding a potentially valuable carbon feedstock [4, 13]. In parallel, catalysts based on metals other than Pd, such as Ag, Au, and Fe, have been reported [14]; however, these systems generally still require harsh operating conditions and an additional H_2_ feed, limiting their practical competitiveness [1, 6, 15, 16, 17, 18, 19]. The key challenge therefore remains the development of less energy‐intensive and operationally milder processes for this important reaction. Electro‐ and photocatalytic approaches (Figure 1) have emerged as promising alternatives to conventional thermal semi‐hydrogenation, offering access to milder and potentially more sustainable reaction conditions.

**FIGURE 1:**
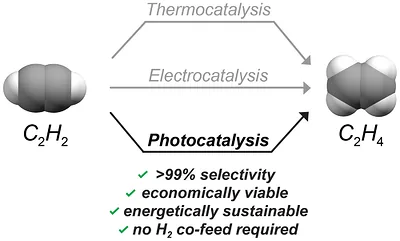
Different catalytic approaches to achieve acetylene semi‐hydrogenation to ethylene, with a focus in this Minireview on emerging photocatalytic methods.

Electrocatalytic acetylene semi‐hydrogenation has gathered significant interest as a room temperature process that uses electricity as the driving force for reduction, thereby avoiding an additional H_2_ feed and harsh reaction conditions. Research into electrochemical acetylene reduction has been going on for decades [20, 21, 22], but has historically been hindered by the low solubility of acetylene in reaction media, sluggish reaction kinetics, and competing H_2_ evolution. Recent advances have begun to address these limitations, with particular emphasis on Cu‐based electrocatalysts [23, 24] supported by computational insights highlighting favorable energetics for acetylene adsorption and ethylene desorption [25]. In this context, reports in 2021 demonstrated that Cu‐based electrodes can effectively drive acetylene semi‐hydrogenation, highlighting the potential of this approach for sustainable ethylene production [7, 25, 26, 27]. Despite these developments, significant challenges remain ahead, including high overpotentials, limited efficiencies, competing H_2_ evolution, and unwanted C─C coupling leading to coke formation.

Standing at the forefront of sustainable ethylene purification is photocatalytic acetylene semi‐hydrogenation (Figure 1), which possibly represents the most elegant and efficient strategy under mild conditions. Unlike the previously mentioned methods, photocatalysis can directly harness sunlight, thereby converting abundant solar energy into chemical energy at room temperature while bypassing the need for external heating or high‐voltage inputs. By using light as a direct energy input, photocatalytic systems can operate under ambient conditions while employing water or organic compounds as hydrogen sources, thereby circumventing the need for molecular H_2_ and the associated requirements for pressurization, dedicated equipment, storage, transport, and handling of flammable gas mixtures [28]. Transfer hydrogenation therefore provides an alternative paradigm in which hydrogen equivalents are generated from alternative donors such as alcohols, formic acid, hydrides, amines, ammonia‐borane, silanes, or water. Such approaches may facilitate controlled hydrogen delivery, operate under milder reaction conditions, and help suppress undesired over‐reduction pathways. The photocatalytic route has shown remarkable selectivity for ethylene production, often reaching near‐unity, and provided remarkable mechanistic insights into acetylene semi‐hydrogenation using well‐defined molecular catalysts. The ability to achieve near‐unity acetylene conversion, without the risk of ethane production or catalyst coking, places photocatalysis in a position of prominence. This Minireview provides a concise and critical overview of this emerging field, placing recent advances within the industrial context of polymer‐grade ethylene production. Emphasis is placed on the mechanistic understanding of acetylene reduction, with a comparative analysis of the three main catalytic pathways reported in the literature. This Minireview also aims to identify current challenges and to establish guidelines and future directions toward the development of next‐generation photocatalytic systems for sustainable ethylene purification.

## 2 Mechanistic Understanding of Photocatalytic Acetylene Semi‐Hydrogenation

Photocatalytic acetylene semi‐hydrogenation reactions predominately rely on multicomponent systems. A multicomponent system usually comprises a catalyst (**CAT**), a light‐harvesting photosensitizer (**PS**), a sacrificial donor (**SD**), and a proton source (**H^+^**). It is noteworthy that some heterogenous systems have been reported to incorporate both the **PS** and **CAT** into the same material, yielding true all‐in‐one photocatalysts. The essential of photocatalytic reactions lies in the conversion of photon energy into chemical energy, thereby driving redox reactions.

Upon light absorption, the **PS** initiates the electron‐transfer sequence that connects the **SD**, the **CAT**, and ultimately acetylene reduction. In molecular systems, excitation of the **PS** generates an excited state (PS*) that can be quenched by either the **SD** or **CAT**, depending on the relative redox potentials of the components (Figure 2a) [29, 30, 31]. In a reductive quenching pathway, electron transfer from the **SD** to PS* affords the reduced **PS^•−^**, which can subsequently deliver an electron to the **CAT**, regenerating ground‐state **PS**. Conversely, in an oxidative quenching pathway, PS* transfers an electron to the **CAT** to generate **PS^•+^**, which is then reduced back to **PS** by the **SD**. The efficiency of these processes is governed by the photophysical and electrochemical properties of the **PS**, including its absorption profile, excited‐state lifetime and energy, and ground‐ and excited‐state redox potentials [32]. The absorption spectrum determines the usable irradiation window, whereas a sufficiently long‐lived excited state is required for productive bimolecular electron transfer to compete with radiative and non‐radiative decay. At the same time, the redox potentials of **PS**, **CAT**, and **SD** must be properly matched to ensure thermodynamically favorable charge‐transfer steps while minimizing unproductive back‐electron transfer. These requirements have motivated the development and use of diverse **PS** classes, including transition‐metal complexes [33], organic dyes, and organic or inorganic semiconductors [34, 35, 36, 37, 38, 39, 40, 41, 42, 43].

**FIGURE 2:**
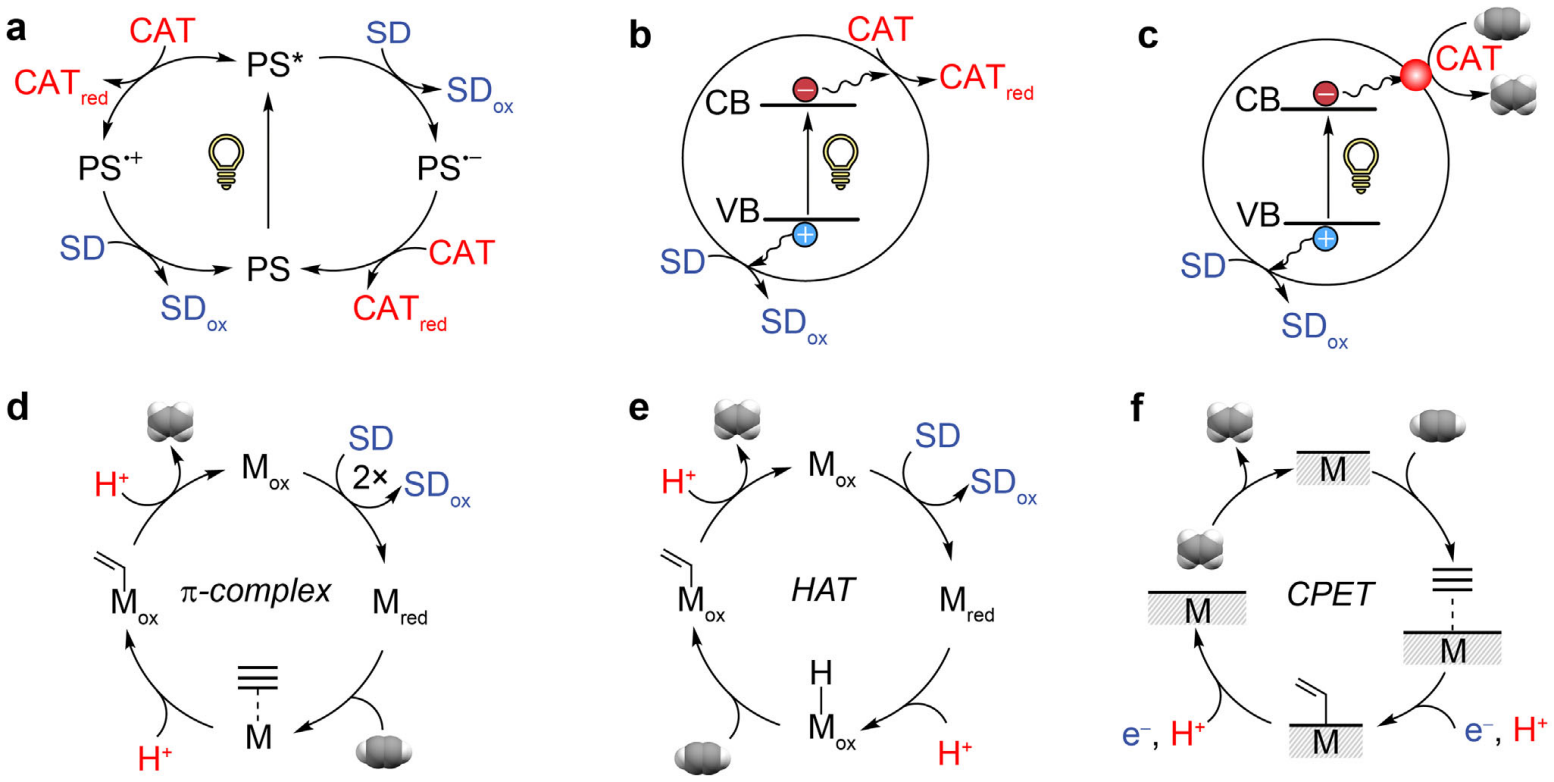
(a) Schematic representation of a homogeneous photosensitizer‐catalyst system and its possible reductive and oxidative quenching pathways. (b) Schematic representation of a heterogeneous semiconductor system. (c) Schematic representation of catalyst‐supported semiconductors system for acetylene semi‐hydrogenation. (d–f) Acetylene semi‐hydrogenation mechanisms mediated by π‐complex pathway (d), or by hydrogen‐atom transfer (HAT) pathway (e), or by concerted proton‐coupled electron transfer (CPET) pathway (f). Abbreviations: CAT = catalysts; PS = photosensitizer; SD = sacrificial donor; VB = valence band; CB = conduction band; M = metal.

In semiconductor‐based systems, light absorption by a material with a suitable bandgap generates electron–hole pairs (Figure 2b) [44, 45]. When charge separation outcompetes recombination, the photogenerated carriers migrate at the semiconductor interface, where electrons drive reduction processes and holes are consumed by oxidation reactions. In the absence of an internal driving force, charge carriers migrate mainly by diffusion, increasing trapping, and recombination losses; however, an excess of **SD** can scavenge photogenerated holes, suppress recombination, and prolong the lifetime of electrons available at the semiconductor surface. Alongside recombination, trapping, and quenching processes [46, 47], the efficiency of photocatalytic reactions is also governed by the density and accessibility of active sites, mass transport limitations, and the intrinsic selectivity of the reaction pathway [48]. An effective photocatalyst should combine stability, low cost, and scalability with strong light harvesting, efficient charge separation, appropriate band‐edge energetics, and abundant reactive sites for catalytic reactions. In general, several strategies have been developed to meet these requirements, including defect engineering, crystal‐facet control, heterojunction construction, and cocatalyst loading [49, 50, 51, 52, 53]. Among them, cocatalyst deposition is particularly effective because it spatially separates light absorption from catalytic turnover: photogenerated electrons are transferred from the semiconductor conduction band (CB) to surface cocatalyst sites, where acetylene reduction occurs (Figure 2c). This architecture improves charge separation, suppresses recombination, and enables independent tuning of the catalytic interface and the light‐harvesting scaffold [54, 55].

In acetylene photocatalytic semi‐hydrogenation, three mechanistic pathways have been reported (Figure 2d–f): π‐complex, hydrogen atom transfer (HAT), and concerted proton‐electron transfer (CPET). In the π‐complex pathway, first described in 2022 [56], a low‐valent metal center coordinates acetylene through metal‐alkyne π‐interaction, driven by the nucleophilic character of the metal center toward the electrophilic nature of the alkyne (Figure 2d). Subsequent protonation of the coordinated acetylene affords hydrogenated intermediate that ultimately evolves into ethylene. The high ethylene selectivity generally observed for this pathway is attributed to the preferential coordination of acetylene over ethylene, consistent with their different electrophilicity [57]. However, when water is used as the proton source, both proton dissociation and protonation of the metal–acetylene π‐complex are kinetically demanding and can become rate‐limiting, leading to only moderate catalytic activity in reported systems [56, 58, 59].

In both chemistry and biology, many processes proceed through proton‐coupled electron transfer (PCET) mechanism [60]. When the proton transfer and electron transfer occur in a single concerted kinetic step, HAT and CPET mechanism can be involved. In the former, a proton and an electron are transferred together as a hydrogen atom from a single donor to a single acceptor; this pathway has been observed in acetylene semi‐hydrogenation through the transfer of hydrogen atom from metal‐hydride (M–H) intermediate to the unsaturated alkyne (Figure 2e) [61, 62, 63, 64, 65, 66, 67]. In general, transition‐metal‐catalyzed HAT (MHAT) has established itself as an effective methodology for the hydrogenation of a variety of organic reactions, including the functionalization of unsaturated C─C bonds [68, 69, 70, 71, 72, 73, 74]. In many systems, the metal center is firstly reduced to form the active low‐valent species and then it is protonated by a suitable proton source to generate the key M–H intermediate. As shown in the pioneering studies by Halpern [75, 76], metal–hydride‐mediated HAT to unsaturated substrates can be described as the formal transfer of a hydrogen atom from M–H to the substrate, generating carbon‐centered radical intermediates that undergo subsequent transformations. In acetylene semi‐hydrogenation, HAT from the metal–hydride to acetylene affords a vinyl radical intermediate, which can be combined with the metal center to form a vinyl–metal species. Subsequent protonation releases ethylene and regenerates the metal, which then can re‐form the M–H. The preference for acetylene semi‐hydrogenation is generally attributed to the favorable interaction between the electrophilic alkyne and the M–H species, together with the rapid interception of the hydride intermediate through HAT [77].

Mechanistic discrimination between π‐complexation and HAT pathways requires combined experimental and theoretical evidence. Hydrogen evolution reaction (HER) is a useful diagnostic because M–H intermediates can also promote H_2_ formation. Thus, the absence of detectable H_2_ under inert conditions supports a π‐complex pathway, whereas in HAT‐mediated C_2_H_2_ reduction, acetylene semi‐hydrogenation and HER could share a common metal–hydride intermediate; accordingly, H_2_ evolution is observed under inert atmosphere, and both reactions show pH‐dependent profiles. Direct evidence for the π‐complex pathway was provided by using in situ surface‐enhanced Raman spectroscopy [59]. Upon irradiation, the ν_(C≡C)_ band red‐shifts, consistent with π‐coordination of acetylene to the metal center within the tetradentate metal–ligand environment, while the subsequent appearance of ν_(C═C)_ features supports ethylene formation. By contrast, radical intermediates are diagnostic of HAT and can be probed by trapping experiments: 2,2,6,6‐tetramethyl‐1‐piperidinyloxy (TEMPO) [61, 63], and trimethyl phosphite (P(OMe)_3_) [66] have been used for this purpose, yielding to either TEMPO‐vinyl adduct or dimethyl vinylphosphonate, which have been detected by LC–MS or GC–MS, respectively. However, because TEMPO may also interact with photogenerated electrons in semiconductors and suppress ethylene formation independently of radical trapping, these effects must be distinguished carefully; unambiguous detection of vinyl‐radical adducts is therefore essential to validate HAT [58]. To date, the available results suggest that systems operating through a HAT mechanism outperform π‐complex pathway in acetylene semi‐hydrogenation. Indeed, HAT has been also applied in electrocatalytic systems for alkyne semi‐hydrogenation [78], where the reaction is also related to the formation of active hydrogen‐atom species that can lead to over‐hydrogenation [79, 80], and competes with HER [81]. However, H_2_ evolution serves as a necessary but not sufficient condition for confirming the HAT pathway. Especially in semiconductor photocatalysts, where H_2_ can also evolve via the direct reduction of protons by photogenerated electrons, a process that is often pronounced in the absence of acetylene.

Alternatively, acetylene semi‐hydrogenation can proceed through a CPET mechanism as recently reported (Figure 2f) [64, 82]. In contrast to HAT, CPET involves the concerted transfer of one electron and one proton to (or from) different reagents. The distinction between HAT and CPET is often subtle and has historically led to ambiguity in mechanistic assignment [83]. Despite several comprehensive reviews addressing this topic [83, 84, 85, 86, 87, 88], unambiguous discrimination between these mechanisms remains challenging, and misinterpretation is still common when relying on limited experimental evidence. However, the detection of H‐atom species could provide a useful diagnostic to distinguish CPET and HAT pathways. The absence of electron paramagnetic resonance (EPR)‐detectable H* species, together with the lack of any effect on the ethylene production upon the addition of 5,5‐dimethyl‐1‐pyrroline *N‐*oxide (DMPO), points to a CPET mechanism in acetylene semi‐hydrogenation [82]. Conversely, the partial suppression of ethylene formation upon the addition of a H* scavenger may indicate the coexistence of both CPET and HAT mechanisms [64]. The two mechanisms are not necessarily mutually exclusive and may even operate sequentially within the same catalytic cycle. Their possible coexistence, or sequential involvement, further complicates their unambiguous identification; therefore, combined experimental and theoretical approaches are essential to support or validate the proposed reaction pathways. Future works should also focus on distinguishing concerted CPET pathways from stepwise ET–PT or PT–ET sequences. This distinction is essential because stepwise mechanisms may involve detectable radicals, reduced, or protonated intermediates, whereas concerted CPET proceeds through a single kinetic step without accumulation of discrete species. However, the detection or non‐detection of reaction intermediates is not, by itself, sufficient to distinguish between HAT and CPET pathways, as intermediate lifetimes depend on the experimental timescale, temporal resolution, and reaction conditions. Reliable mechanistic assignment will therefore require converging electrochemical, spectroscopic, kinetic isotope effects, pH‐dependent effects, and computational evidence rather than reliance on any single diagnostic experiment.

## 3 Catalysts for Acetylene Semi‐Hydrogenation

In recent years, significant efforts have been devoted to the development of photocatalytic systems for the selective semi‐hydrogenation of acetylene to ethylene. This section provides an overview of both homogeneous and heterogeneous approaches reported to date, highlighting their distinct mechanistic features, advantages, and limitations in comparison to the conventional thermocatalytic process.

### 3.1 Homogeneous Catalysts

By operating in the same phase as the reactants, homogeneous catalysts decrease the activation energy by directly binding the substrate and stabilizing reactive intermediates. Their well‐defined and tunable active sites make molecular systems particularly valuable for mechanistic studies and in situ characterization [89], although catalyst recovery and recyclability remain important limitations. To date, all homogeneous systems reported for photocatalytic acetylene semi‐hydrogenation are based on cobalt catalysts and operate through one of two pathways: π‐complex formation between low‐valent cobalt species and acetylene, or HAT mediated by Co–H intermediates.

The first photocatalytic system, reported by our groups in 2022, combined water‐soluble Co(III) porphyrin **CAT**s (CoTPPS or CoTCPP, Table 1, entry 1, Figure 3a) with tris(2,2′‐bipyridyl)dichlororuthenium(II) hexahydrate [Ru(bpy)_3_]^2+^ or mesoporous graphitic carbon nitride (mpg‐CN) (Figure 3b) as **PS**s and ascorbic acid (AscH) or triethanolamine (TEOA) as **SD**s (Figure 3c) [56]. The system proceeds via a π‐complex pathway involving a nucleophilic Co(I) intermediate, generated through sequential chemical reduction by AscH and photochemical electron‐transfer steps. Remarkably, the system achieved a turnover number (TON) of 1967 in 24 h in pure acetylene atmosphere and near 100% conversion and ≥99% selectivity for ethylene under industrially relevant conditions over 28 h of irradiation, using water as the proton source. Mechanistic studies, including pH‐dependent and radical trapping experiments, ruled out the involvement of Co–H intermediates and supported the π‐complex mechanism.

**TABLE 1:** Catalytic performances of homogeneous photocatalytic systems for the reduction of acetylene to ethylene in batch.

Entries	Photocat. mixture	Irradiation	Performance under pure C_2_H_2_	Performance under C_2_H_4_ co‐feed	Refs.
1	CoTPPS or CoTCPP (CAT) [Ru(bpy)_3_]^2+^ (PS) NaAsc (SD) NaHCO_3_ buffer H_2_O (H^+^)	450 nm LED	CoTPPS TON_C2H4_ = 1967 (24 h)^a^ AQY (450 nm) = 0.11%^a^ S_C2H4_ = 99.4%^a^	CoTPPS C_2_H_4_ Production Cycle = 28 h^d^ Residual C_2_H_2_ <5 ppm S_C2H4_ = 98.9%^d^	[56]
2	[Co(N_4_S_2_)]^2+^ (CAT) CuPS‐SO_3_Na (PS) NaAsc (SD) NaHCO_3_ buffer H_2_O (H^+^)	300 W Xe lamp (*λ* > 420 nm)	TON_C2H4_ = 323 (15 h)^b^ AQY (380 nm) = 0.05%^b^ S_C2H4_ = 97.1%^b^	C_2_H_4_ Production Cycle = 15 h^e^ Residual C_2_H_2_ <1% S_C2H4_ = 97.6%^e^	[59]
3	Co(dmgH)_2_PyCl (CAT) [Ru(bpy)_3_]^2+^ (PS) BIH (SD) TFE (H^+^) CH_3_CN	450 nm LED	TON_C2H4_ = 1148 (20 h)^a^ AQY (450 nm) = 1.0%^a^ S_C2H4_ > 99.9%^a^	C_2_H_4_ Production Cycle = 50 min^d^ Residual C_2_H_2_ <5 ppm S_C2H4_ > 99.9%^d^	[63]
4	Co(dmgH)_2_ImHCl (CAT) CuPS (PS) TEA (SD, H^+^) H_2_O (H^+^):CH_3_CN (1:1 *v/v*)	405 nm LED	TON_C2H4_ = 670 (48 h)^a^ AQY = n.r. S_C2H4_ = 99.6%^a^	C_2_H_4_ Production Cycle = 105 min^d^ Residual C_2_H_2_ <5 ppm S_C2H4_ = 99.3%^d^	[67]
5	Cosalen or Cosalen‐5‐OMe (CAT) [Ru(dmb)_3_]^2+^ (PS) BIH (SD) DMF:TEOA:H_2_O (H^+^) (7:2:1 *v/v*)	450 nm LED	Cosalen‐5‐OMe TON_C2H4_ = 29401 (10 h)^c^ S_C2H4_ = 99.9%^c^ Cosalen AQY (450 nm) = 1.41%^c^	Cosalen C_2_H_4_ Production Cycle = 8 h^f^ Residual C_2_H_2_ = 3.7 ppm S_C2H4_ = 99.0%^f^	[66]

^a^ ≥99.5 vol% C_2_H_2_.

^b^ 1.0 vol% C_2_H_2_, Ar balance.

^c^ ≥97.5 vol% C_2_H_2_.

^d^ 1.0 vol% C_2_H_2_, 30.0 vol% C_2_H_4_, He balance.

^e^ 0.2 vol% C_2_H_2_, 20.0 vol% C_2_H_4_, He balance.

^f^ 1.1 vol% C_2_H_2_ in C_2_H_4_; n.r. = not reported; CAT = catalyst; PS = photosensitizer; SD = sacrificial donor; H^+^ = proton donor; TON = turnover number; AQY = apparent quantum yield.

**FIGURE 3:**
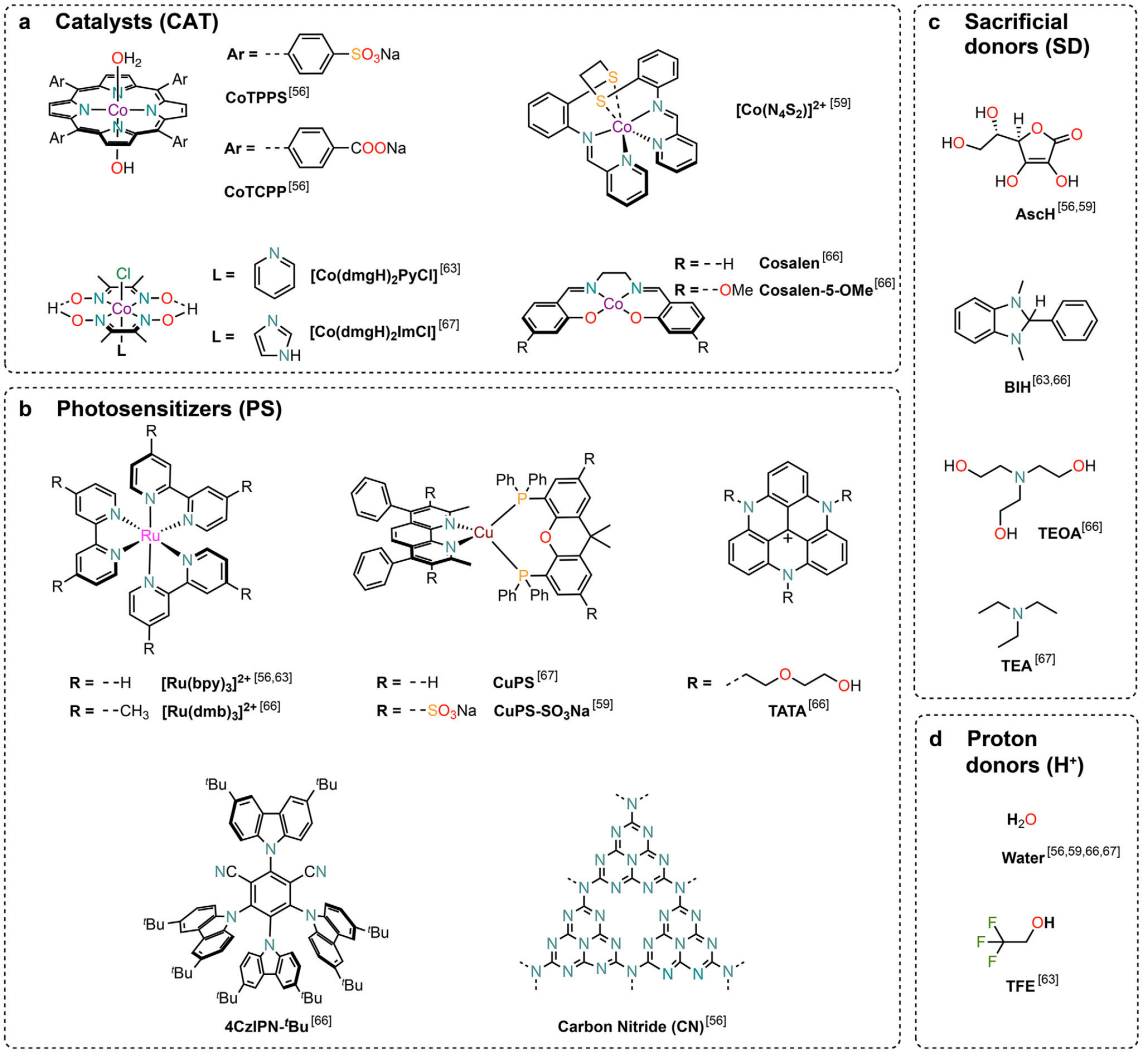
Main reported components of homogeneous photocatalytic mixtures for the acetylene semi‐hydrogenation to ethylene. (a) Homogeneous catalysts (**CAT**). (b) Organic and inorganic photosensitizers (**PS**). (c) Sacrificial donors (**SD**). (d) Proton sources (H^+^).

A second π‐complex system was reported in 2025 using a bioinspired cobalt complex featuring an N_4_S_2_ coordination environment inspired by nitrogenase active sites (Table 1, entry 2, Figure 3a) [59]. This ligand environment increases the nucleophilicity of the Co(I) center, thereby promoting acetylene activation. When coupled with a Cu‐based **PS** (CuPS‐SO_3_Na, Figure 3b), the system achieved acetylene conversion to ethylene, including 99% conversion in the presence of an ethylene co‐feed, with 97.6% selectivity under mild aqueous conditions. The weak interaction between ethylene and the cobalt center favors product desorption and suppresses over‐hydrogenation.

In parallel, homogenous systems operating through HAT pathway have been developed [77]. Although Co–H intermediates are well‐established in hydrogen evolution [90, 91], and alkyne reduction catalysis [92, 93, 94], their use in homogeneous photocatalytic acetylene semi‐hydrogenation was exploited by us in 2025 with a cobaloxime **CAT** (Table 1, entry 3, Figure 3a) [63]. The cobaloxime‐based photocatalytic system reached a TON of 1148 under pure acetylene atmosphere and enabled a 30‐fold faster conversion rate compared to the CoTPPS system, achieving complete acetylene conversion within 50 min of irradiation while maintaining ≥99.9% ethylene selectivity in an industrially relevant mixture. Radical‐trapping experiments confirmed the HAT pathway through LC–MS detection of vinyl‐radical intermediates. This cobaloxime platform was subsequently improved through two complementary design strategies (Table 1, entry 4): increasing the electron‐donating ability of the axial ligand and replacing the Ru‐based **PS** with a Cu‐based one (CuPS) (Figure 3b) [67]. The more electron‐rich ligand facilitates formation and reactivity of the Co–H intermediate by stabilizing low‐valent cobalt species and promoting protonation steps. In parallel, substitution of [Ru(bpy)_3_]^2+^ with a Cu‐based **PS** afforded a fully noble‐metal‐free system and increased ethylene production by 3‐fold relative to the Ru‐based analogue.

The performance of hydride‐based systems was further advanced using Cosalen complexes (Table 1, entry 5, Figure 3a), in which ligand electronics were also exploited to accelerate Co–H formation and HAT reactivity [66]. In this system, Cosalen operates with tris(4,4'‐dimethyl‐2,2'‐bipyridine)ruthenium(II) ([Ru(dmb)_3_]^2+^) **PS** (Figure 3b) and 1,3‐dimethyl‐2‐phenyl‐2,3‐dihydro‐1*H*‐benzo[*d*]imidazole (BIH) **SD** under visible‐light irradiation (Figure 3c), reaching >99.9% ethylene selectivity and high TON values under pure acetylene, with negligible side‐products. Mechanistic studies supported a Co–H‐mediated HAT pathway: photogenerated [Ru(dmb)_3_]^2+^ reduces Co^II^ to Co^I^, protonation by water affords Co^III^–H, and HAT to acetylene generates a vinyl radical that collapses to a Co–vinyl intermediate before protonation and electron transfer yields ethylene. Importantly, increasing the electron density of the salen ligand facilitates Co^III^–H formation, as shown by the 5‐OMe‐substituted Cosalen CAT derivative, which reached a TON of 29401 in 10 h while maintaining near‐unity selectivity.

Overall, homogeneous systems have established key mechanistic paradigms for photocatalytic acetylene semi‐hydrogenation, demonstrating that both π‐complex and HAT pathways can achieve high activity and selectivity. However, further advances will be required before homogeneous photocatalytic systems can be considered viable for large‐scale industrial deployment, including improved catalyst recovery and stability, implementation under continuous‐flow conditions, and the development of catalysts based on more earth‐abundant elements. Ideally, the mechanistic knowledge gained from these molecular systems should also guide the design of benchmark heterogeneous semiconductor photocatalysts that operate without **SD**s, thereby moving the field toward more practical and scalable architectures.

### 3.2 Porous Organic Framework Heterogeneous Catalysts

While homogeneous systems have demonstrated remarkable activity and selectivity in acetylene semi‐hydrogenation, their practical implementation is limited by catalyst deactivation, limited long‐term stability, and the lack of straightforward recovery and recycling strategies. In this context, heterogeneous **CAT**s based on porous frameworks or semiconductor scaffolds offer greater robustness, easier separation, and improved operational stability. These features, together with the possibility of engineering confined active sites and integrated architectures, make porous frameworks attractive heterogeneous platforms for bridging molecular‐level mechanistic control and practical application. Moreover, because several molecular systems still rely on dissolved **PS**s, process integration and scale‐up remain challenging, motivating the development of all‐in‐one materials that combine light absorption and catalytic function within a single unit. Furthermore, several important questions remain open for porous photocatalytic systems. In particular, it is often unclear whether catalysis occurs inside the pores or predominantly at the external surface of the material. Although gas‐phase sorption measurements with acetylene and ethylene are sometimes reported, they do not necessarily reflect the affinity of a porous material for either gas under solution‐phase photocatalytic conditions. Solvation, competitive solvent adsorption, pore accessibility, and gas partitioning can substantially alter the relative uptake of C_2_H_2_ and C_2_H_4_ compared with the gas phase. For this reason, gas‐phase sorption isotherms should be interpreted with caution when discussing the photocatalytic behavior of materials dispersed in solution.

The design of the host framework is central to controlling catalytic performance. Early heterogeneous approaches focused on incorporating cobalt porphyrins (CoTCPP) as linkers in metal–organic frameworks (MOFs), thereby creating robust materials with spatially isolated, “single‐site” catalytic centers [95]. A representative example is Co‐PCN‐222 (Table 2, entry 1, Figure 4a) [61], in which the chemically stable Zr‐based framework enabled 7‐fold longer catalytic operation while maintaining a remarkable 99.9% selectivity for C_2_H_4_ under industrially relevant conditions. Building on the well‐established efficiency of cobaloximes for electrocatalytic hydrogen evolution (HER) [96], this molecular catalyst platform was subsequently incorporated into MOF‐based architectures for photocatalytic acetylene reduction (Table 2, entry 2, Figure 4b) [63]. The system retained comparable high selectivity while markedly accelerating acetylene‐to‐ethylene conversion in C_2_H_4_ co‐feed and improved stability relative to the porphyrin‐based analogue. However, these bulk MOF architectures can still suffer from limited aqueous stability, mass‐transport constraints, and reliance on organic solvents.

**TABLE 2:** Catalytic performances of heterogeneous photocatalytic systems containing porous organic frameworks catalysts for the reduction of acetylene to ethylene in batch.

Entries	Photocat. mixture	Irradiation	Performance under pure C_2_H_2_	Performance under C_2_H_4_ co‐feed	Refs.
1	Co‐PCN‐222 (CAT) [Ru(bpy)_3_]^2+^ (PS) TEOA (SD and H^+^) CH_3_CN	450 nm LED	C_2_H_4_ Production = 1600 mmol g^−1^ Co^a^ AQY (450 nm) = 0.02% Durability = 168 h^a^ Recyclability = 5 cycles of 4 h each^a^ S_C2H4_ = 99.7%^a^	C_2_H_4_ Production Cycle = 87 h^d^ Residual C_2_H_2_ <5 ppm S_C2H4_ > 99.9%^d^	[61]
2	UU‐100(Co) (CAT) [Ru(bpy)_3_]^2+^ (PS) BIH (SD) TFE (H^+^) CH_3_CN	450 nm LED	C_2_H_4_ Production = 698 mmol g^−1^ Co^a^ AQY = n.r. Durability = n.r S_C2H4_ > 99.9%^a^	C_2_H_4_ Production Cycle = 4 h^d^ Residual C_2_H_2_ <5 ppm Recyclability = 6 cycles of 8 h each^d^ S_C2H4_ > 99.9%^d^	[63]
3	Cu‐Co‐MNSs (CAT) [Ru(bpy)_3_]^2+^ (PS) NaAsc (SD) NaHCO_3_ buffer (H_2_O, H^+^)	450 nm LED	C_2_H_4_ Production = 3.61 mmol g^−1^ Co^b^ AQY = n.r. Durability = 12 h^b^ Recyclability = n.r. S_C2H4_ = 99.5%^b^	C_2_H_4_ Production Cycle = 68 h^e^ Residual C_2_H_2_ <5 ppm S_C2H4_ = 90.3%^e^	[62]
4	CoO_4_‐ or CoN_4_‐MOF (CAT) [Ru(bpy)_3_]^2+^ (PS) TEA (SD) H_2_O (H^+^):CH_3_CN	200 mW cm^−2^ Xe lamp (420 < λ < 800 nm)	CoO_4_‐MOF C_2_H_4_ Production = 0.85 mmol g^−1^ h^−1^ ^c^ AQY = n.r. Durability = 14 h^c^ Recyclability = 6 runs of 4 h each^c^ S_C2H4_ = traces of C_2_H_6_ ^c^	CoN_4_‐MOF C_2_H_4_ Production Cycle = 70 h^f^ Residual C_2_H_2_ <0.1% S_C2H4_ = n.r.	[97]
5	Cz‐Co‐COF‐H (CAT) [Ru(bpy)_3_]^2+^ (PS) NaAsc (SD) H_2_O (H^+^):CH_3_CN (1:1 *v/v*)	300 W Xe Lamp	C_2_H_4_ Production = 1.76 mmol g^−1^ h^−1^ ^a^ AQY = n.r. Durability = 12 h^a^ Recyclability = 5 run^a^ S_C2H4_ = traces of C_2_H_6_ ^a^	C_2_H_4_ Production Cycle = 96 h^d^ Residual C_2_H_2_ = 0.08% S_C2H4_ = traces of C_2_H_6_ ^d^	[65]

^a^ ≥99.5 vol% C_2_H_2_.

^b^ ≥97.5 vol% C_2_H_2_.

^c^ 99.9 vol% C_2_H_2_.

^d^ 1.0 vol% C_2_H_2_, 30.0 vol% C_2_H_4_, He balance.

^e^ 1.1 vol% C_2_H_2_ in C_2_H_4_

^f^ 1.0 vol% C_2_H_2_ in C_2_H_4_; n.r. = not reported; CAT = catalyst; PS = photosensitizer; SD = sacrificial donor; H^+^ = proton donor; AQY = apparent quantum yield.

**FIGURE 4:**
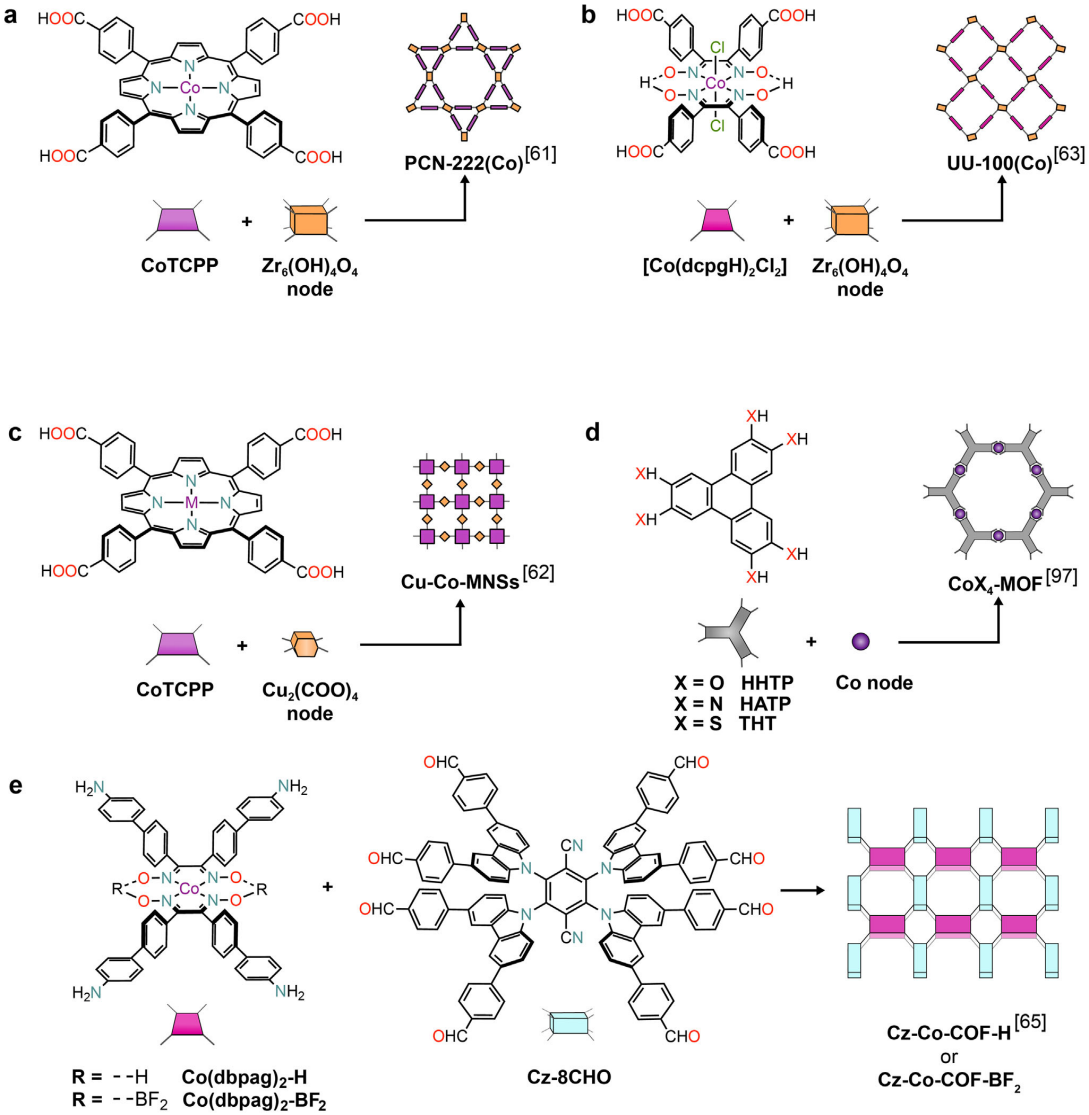
Design concept of various porous organic framework heterogeneous catalysts reported to date. (a) The PCN‐222(Co) MOF composed of the CoTCPP porphyrin **CAT** and the Zr_6_(OH)_4_O_4_ nodes [61]. (b) The UU‐100(Co) MOF composed by [Co(dcpgH)_2_Cl] cobaloxime **CAT** and the Zr_6_(OH)_4_O_4_ nodes [63]. (c) The Cu‐Co‐MNSs MOF composed by the CoTCPP porphyrin **CAT** and Cu(COO)_4_ nodes [62]. (d) The CoX_4_‐MOFs composed by HHTP/HATP/THT ligands bonded to Co node [97]. (e) The Cz‐Co‐COF‐H and Cz‐Co‐COF‐BF_2_ composed by Cz‐8CHO unit and Co(dbpag)_2_‐H or Co(dbpag)_2_‐BF_2_ cobaloximes **CAT**, respectively [65].

Two‐dimensional (2D) MOFs have been proposed as a strategy to address some of these limitations by increasing substrate accessibility and shortening diffusion pathways. For example, nanosheet MOFs constructed from Co porphyrin linkers (CoTCPP) and Cu paddle‐wheel nodes (Table 2, entry 3, Figure 4c) showed good performance under aqueous conditions, with water acting as both solvent and proton source, reaching 90.3% semi‐hydrogenation selectivity under industrially relevant conditions [62]. Beyond improving accessibility, 2D architectures also enable systematic tuning of the local coordination environment. Triphenylene‐based 2D Co‐MOFs bearing O, N, or S donor atoms (e.g. CoO_4_, CoN_4_, and CoS_4_, Table 2, entry 4, Figure 4d) revealed that ligand identity modulates reaction barriers, gas adsorption, and charge migration [97]. In this series, CoO_4_ performed best under pure acetylene, whereas CoN_4_ gave higher conversion under ethylene‐rich conditions, highlighting how electronic structure at the metal center controls both intermediate stabilization and competitive substrate adsorption. However, the stability of these materials remains insufficient, particularly under photocatalytic conditions in aqueous media, indicating that further improvements in framework robustness are required before such systems can be considered for practical application.

Although MOFs have dominated framework‐based Co catalysis, covalent organic frameworks (COFs) are emerging as attractive alternatives because of their high stability, large surface area, and greater chemical robustness especially in aqueous conditions. Glyoximate‐based COFs (Table 2, entry 5, Figure 4e) provide a clear example of how the local chemical environment can direct the reaction pathway [65]. In a series of water‐compatible Cz‐M‐COFs (M = Co, Mn, and Ni), tuning both the metal center and terminal ligand environment showed that Cz‐Co‐COF‐H delivers the best performance. This activity was attributed to O–H–O groups that act as potential proton relays, facilitating water dissociation and lowering the free‐energy barrier for acetylene adsorption, thereby promoting subsequent hydrogenation through a proposed HAT mechanism.

### 3.3 All‐in‐One Photocatalysts

Despite the advances discussed so far, these framework‐based catalysts still rely on Ru‐based **PS**s, whose cost, limited abundance, and susceptibility to photodegradation remain important bottlenecks [98, 99]. Moreover, the use of molecular dyes complicates recycling of the photocatalytic systems, as both the **CAT** and the **PS** should be recovered. These limitations have driven the development of integrated “all‐in‐one” materials, in which light harvesting and catalysis are combined within a single material platform, enabling the simultaneous recovery of both components.

In this context, carbon nitride (CN)‐based materials are attractive semiconductors that can act as support for the anchoring of both metal clusters [64, 82] and single‐atom catalysts [58, 100], thus providing versatile photocatalytic platforms in general. For acetylene semi‐hydrogenation, Co‐based CN (Table 3, entry 1, Figure 5a) exhibited exceptional durability, operating for up to 960 h [58], outperforming previously reported photocatalysts in long‐term stability. Atomically dispersed Co sites enabled near‐unity selectivity and high ethylene production (390 mmol g^−1^ Co) under visible‐light irradiation under acetylene and in pure water; however, the reaction proceeded through a π‐complex pathway rather than a Co–H intermediate, limiting the overall kinetics. Subsequent studies showed that introducing nitrogen vacancies can significantly accelerate acetylene reduction [100], while heteroatom doping, as in S‐doped CN supporting Pd species, provides an additional handle to tune activity and selectivity under simulated sunlight (Table 3, entry 2, Figure 5a) [64].

**TABLE 3:** Catalytic performances of heterogeneous photocatalytic systems containing all‐in‐one photocatalysts for the reduction of acetylene to ethylene in batch.

Entries	Photocat. mixture	Irradiation	Performance under pure C_2_H_2_	Performance under C_2_H_4_ co‐feed	Refs.
1	Co@CN TEOA (SD) H_2_O (H^+^)	405 nm LED	C_2_H_4_ production = 390 mmol g^−1^ Co^a^ AQY = n.r. Durability = 960 h^a^ Recyclability = 5 cycles of 4 h each^a^ S_C2H4_ >99.9%^a^	n.r.	[58]
2	Pd/CN or Sulfurized Pd/CN HCl (H^+^) DMF	300 W Xe lamp (300 < λ < 780 nm) or solar	Sulfurized Pd/CN C_2_H_4_ production = ca. 0.42 mmol (5 h)^b^ AQY = n.r. Durability = n.r. S_C2H4_ = 93% Pd/CN AQY (350 nm) = 0.71%	n.r.	[64]
3	Ni(OH)_2_/CN‐OH TEOA (SD, H^+^) H_2_O	300 W Xe lamp (λ > 320 nm)	C_2_H_4_ production = 15.7 mmol g^−1^ h^−1^ ^c^ AQY (365 nm) = 2.36% Durability = 10 h^c^ Recyclability = 5 cycles of 8 h each^c^ S_C2H4_ = 98.2%^c^	C_2_H_4_ production cycle = 1.83 h^e^ Residual C_2_H_2_ <5 ppm S_C2H4_ = n.r.	[82]
4	Pd/TiO_2_ MeOH (SD) H_2_O (H^+^)	300 W Xe lamp	C_2_H_4_ Production = 4.47 mmol g^−1^ h^−1^ ^d^ AQY (350 nm) = 3.8% Durability = 6 h^d^ S_C2H4_ = 77.4%^d^	n.r.	[101]
5	Cu@HOF HAsc (SD) H_2_O (H^+^)	450 nm LED	C_2_H_4_ production = 3010 mmol g^−^^1^ Cu^a^ AQY = n.r. Durability = 16 h^a^ S_C2H4_ >99.9%^a^	C_2_H_4_ production cycle = 75 min^f^ Residual C_2_H_2_ <5 ppm Recyclability = 4 production cycles^f^ S_C2H4_ = 99.8%^f^	[102]

^a^ ≥99.5 vol% C_2_H_2_.

^b^ 0.42 mmol (4.0 vol%) C_2_H_2._

^c^ 99.9 vol% C_2_H_2_.

^d^ 5.0 vol% C_2_H_2_, Ar balance.

^e^ 1.0 vol% C_2_H_2_ in C_2_H_4_.

^f^ 1.0 vol% C_2_H_2_, 30.0 vol% C_2_H_4_, N_2_ balance; n.r. = not reported; SD = sacrificial donor; H^+^ = proton donor; AQY = apparent quantum yield.

**FIGURE 5:**
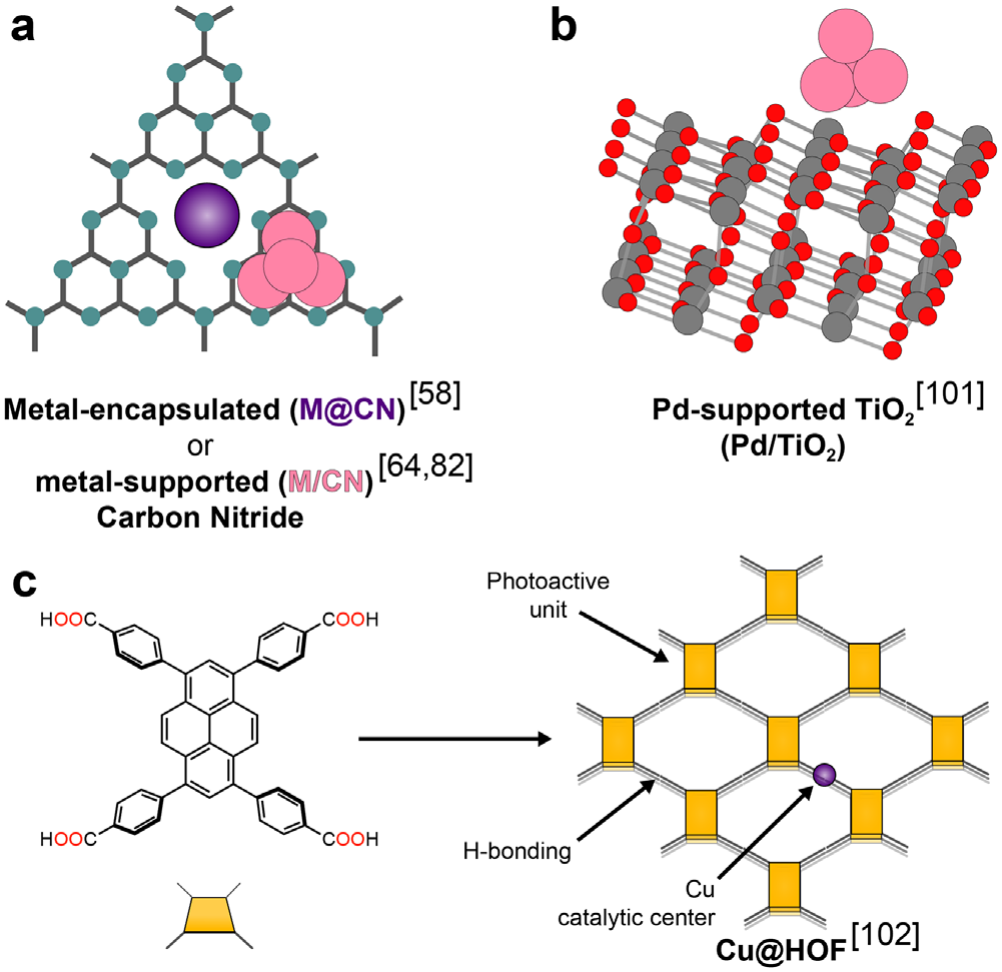
Schematic representation of structures of all‐in‐one photocatalysts reported to date. (a) Metal‐encapsulated or ‐supported Carbon Nitride (M@CN or M/CN, respectively) [58, 64, 82, 100]. (b) Pd‐supported TiO_2_ (Pd/TiO_2_) [101]. (c) Copper‐loaded hydrogen‐bonded organic framework based on 1,3,6,8‐tetrakis(*p*‐benzoic acid)pyrene (Cu@HOF) [102].

In CN‐based photocatalysts, performance can be further improved by engineering the local proton‐transfer environment at the semiconductor‐cocatalyst interface [82]. Hydroxyl‐modified CN materials bearing Ni(OH)_2_ active sites were hypothesized to promote hydrogen‐bonding interactions with TEOA (**SD**) mediated by interfacial water, facilitating its donor deprotonation and improving electron transfer to the catalytic sites (Table 3, entry 3, Figure 5a). This interfacial proton relay was proposed to enable CPET during acetylene reduction, thereby improving semi‐hydrogenation efficiency. More broadly, this example shows that activity is governed not only by the identity of the metal center, but also by the local environment that controls proton delivery, charge transfer, and substrate activation.

Beyond CN‐based systems, electronic metal–support interactions have also emerged as a key factor in controlling photocatalytic behavior. In Pd/TiO_2_ systems (Table 3, entry 4, Figure 5b), these interactions generate an interfacial electric field that promotes charge transfer from the semiconductor to the metal sites, regulates surface hydrogen species (H*) and facilitates C_2_H_4_ desorption, thereby suppressing competing H_2_ evolution and enhancing semi‐hydrogenation activity [101].

Alongside these semiconductor platforms, hydrogen‐bonded organic frameworks (HOF) have recently emerged as promising all‐in‐one photocatalysts, combining synthetic simplicity, modularity, and facile metal incorporation. The first Cu‐based HOF photocatalyst for acetylene semi‐hydrogenation was recently reported (Table 3, entry 5, Figure 5c), in which photoexcitation generates charge carriers that promote Cu–H formation through water activation, enabling acetylene reduction via a HAT pathway [102]. This system achieved high activity and near‐quantitative selectivity, including 99.8% selectivity under industrially relevant conditions, highlighting HOFs as sustainable and structurally versatile alternatives to more established heterogeneous platforms. The Cu@HOF is also a rare example of a porous scaffold that does not rely on an external **PS** but instead incorporates the photoactive unit within the framework; this may contribute to its relatively high activity.

## 4 Toward Practical Implemental: Flow Photocatalysis and Techno‐Economic Analysis

Industrial implementation requires photocatalytic systems that are not only selective and active, but also reliable under continuous operation. In particular, production efficiencies above 10 L g^−1^ h^−1^ have been proposed as a benchmark for industrial relevance [63]. Batch studies have shown promising performance under industrially relevant feeds, where acetylene is present as a minor impurity (∼1 vol%) in excess ethylene. However, translation beyond batch operation remains challenging, mainly because scale‐up in photocatalysis is limited by light attenuation in large reaction volumes. Flow reactors provide an effective strategy to overcome this limitation by reducing optical path lengths, improving irradiation homogeneity, and enhancing mass transfer [103, 104].

Homogeneous systems can be adapted to flow by combining the gaseous feed with a liquid stream containing the photocatalytic mixture. In the Cosalen‐5‐OMe system, crude ethylene containing 1.10 vol% acetylene was introduced into an irradiated capillary reactor equipped with 460 nm LEDs, where it formed a stable slug flow with the catalytic solution [66]. This setup, composed of liquid slugs and elongated gas bubbles, enhances gas–liquid mass transfer, improves light distribution, and increases photon flux within the thin liquid film surrounding the bubbles. Internal circulation within the slugs continuously renews this irradiated film, maintaining efficient exposure of the reaction mixture to light. Under these conditions, acetylene was reduced to undetectable levels within 38 min, corresponding to a 13‐fold improvement over batch operation, while polymer‐grade ethylene was produced with >99.9% selectivity for more than 50 h. The photocatalytic mixture could also be reused at least three times while still meeting the conversion required for industrial application. The system achieved a production rate of 135 mL h^‒1^ and was scaled by at least a factor of 10 without loss of selectivity, with further scale‐up possible through internal or external numbering‐up and reactor enlargement [66, 104].

Gas‐feed rate is another critical parameter in flow photocatalysis, because it directly controls residence time and therefore conversion. This was demonstrated for the Pd/mpg‐CN photocatalyst under continuous‐flow conditions [64]. At 2 standard cubic centimeters per minute (sccm), the system achieved >93% conversion and >92% selectivity using a 1.0 vol% acetylene feed in Ar. In a crude ethylene stream containing 0.5 vol% C_2_H_2_ and 20 vol% C_2_H_4_ in Ar, it sustained near‐quantitative acetylene conversion, remaining >99% for over 72 h. Increasing the flow rate progressively decreased acetylene conversion because of the shorter residence time, but this limitation could be mitigated by increasing reactor volume or connecting reactors in series. Expanding the photoreactor from 40 to 250 mL maintained near‐quantitative conversion up to 8 sccm, whereas at 32 sccm conversion dropped to ∼60% in a single reactor but was restored to 94% using two reactors in series, while preserving high selectivity (>94%). These results identify residence time and reactor design as key parameters for increasing throughput without compromising selectivity. Under more concentrated conditions, representative of coal‐derived acetylene feeds (35 vol% C_2_H_2_ in Ar), the system reached 40% conversion at 2 sccm with selectivity >92%, corresponding to an ethylene production rate of 0.60 mmol h^−1^.

Beyond reactor engineering, industrial scale‐up requires rigorous techno‐economic analysis [105]. At present, however, such studies remain very limited, with only one available techno‐economic assessment for photocatalytic acetylene hydrogenation, namely that reported for the Pd/mpg‐CN system [64]. This study indicates that photocatalysis can reduce operating costs by replacing part of the energy demand associated with thermocatalytic processes with light, ideally from renewable sources. The successful implementation of direct solar irradiation further suggests a pathway toward reducing, or even eliminating, the need for grid‐derived electricity for photon generation, thereby improving the overall energy efficiency of the process.

For ethylene purification from naphtha cracking gas, the techno‐economic analysis compared the Pd/mpg‐CN photocatalytic system with the conventional three‐stage thermocatalytic process commonly used in industry, based on a Pd/Al_2_O_3_‐type catalyst for tail‐end hydrogenation [64]. In the thermocatalytic case, the cost structure is overwhelmingly dominated by utility consumption. Utility cost accounts for 94.92% of the production cost, whereas fixed capital, working capital, raw materials, and consumables each contribute only marginally. This confirms that the thermocatalytic route is economically penalized primarily by the energy demand associated with heating, steam, and related process utilities. By contrast, in the photocatalytic case the dominant contribution is not utility consumption but the Pd‐containing photocatalyst itself. For the Pd/mpg‐CN system applied to ethylene purification from naphtha cracking gas, no **SD** is used and the catalyst accounts for 98.81% of the photocatalytic acetylene‐conversion cost. Even after lowering the Pd loading to 1.0 wt.%, the catalyst still contributes 96.15% of the total, whereas utility cost falls to only 2.42%. Under these assumptions, the Pd/mpg‐CN photocatalytic system becomes economically comparable to the thermocatalytic benchmark when the acetylene conversion reaches 86%, that is, when the unit cost of photocatalytic conversion approaches the fixed benchmark cost of the thermocatalytic process.

Taken together, these results reveal a clear distinction between the two approaches. Thermocatalysis is mainly burdened by utility demand, whereas the current Pd/mpg‐CN photocatalytic system is mainly limited by catalyst cost. The economic case for photocatalytic ethylene purification will therefore depend less on marginal improvements in selectivity alone and more on achieving high conversion with cheaper catalyst formulations, ideally based on non‐noble metals, while retaining the intrinsic advantages of mild conditions and the avoidance of additional supply of gaseous H_2_. Such developments could strengthen the key advantages of photocatalysis over both thermocatalytic and electrocatalytic approaches by reducing the contribution of energy costs to the overall process economics. In this sense, photocatalysis may directly address one of the major production‐cost limitations of thermocatalysis, namely the large contribution of electricity and steam demand.

Importantly, the same techno‐economic analysis also indicates that the photocatalytic acetylene hydrogenation approach may be relevant not only to ethylene purification but also to ethylene production. In that study, an industrial‐scale process was designed and simulated in which coal is first converted to acetylene by hydrogen plasma technology and then to ethylene by photocatalytic acetylene hydrogenation. Based on cost and sensitivity analyzes, a maximum Pd loading of 3.0 wt.% and a minimum catalyst lifetime of 1.5 years were identified as the conditions under which photocatalytic ethylene production would remain profitable across a broad range of ethylene market prices, thus defining a relatively large profitable operating window.

The feasibility and advantages of alternatives to conventional thermocatalytic systems are also reflected in recent techno‐economic evaluations of electrocatalytic acetylene semi‐hydrogenation, which provide important economic considerations that can also guide the development of photocatalytic systems. These recent electrocatalytic approaches are generally based on copper‐containing catalysts, and their techno‐economic analyses indicate that electrocatalytic acetylene semi‐hydrogenation, particularly when water is used as the proton source and renewable electricity as the energy input, could provide petroleum‐independent route both for industrial ethylene synthesis from acetylene derived from natural gas or coal [106, 107, 108] and for ethylene purification from acetylene contaminants [109]. These analyses indicate that industrial feasibility relies on simultaneously meeting stringent requirements in terms of selectivity, productivity, energy efficiency and long‐term operational durability. More specifically, they provide several economic considerations that can continue to guide not only electrocatalytic but also photocatalytic approaches. First, the use of renewable energy is essential to remove what would otherwise be the most important cost contributor and to ensure that the CO_2_ emissions associated with coal‐ and natural‐gas‐derived ethylene remain lower than those of the petroleum route [106]. Second, separation‐related costs can account for up to 17.6% of the total process cost [106], meaning that suppression of ethane and competing hydrogen evolution is essential to maintain efficiency under high‐throughput conditions. Third, further advances in the technologies used to produce acetylene feedstock from coal and natural gas will improve the economic viability of coal‐ and natural‐gas‐derived ethylene strategies [108]. Fourth, catalyst design and optimization lower the threshold of the performance parameters required to make the process profitable [107, 108] with catalyst stability emerging as a major factor. For example, the replacement of a Cu‐based electrocatalyst operating with 80% Faradaic efficiency at a current density of 20 mA cm^−2^ accounts for 43% of the total cost of 1.36$ per kg of acetylene, whereas the corresponding treatment cost for the thermocatalytic process is 1.74$ per kg of acetylene under operating conditions of 10 bar and 150°C with 75% heating efficiency, where the primary cost drivers are temperature and pressure [109].

Taken together, these considerations establish key design criteria for next‐generation photocatalysts, namely high activity at low metal loading, long operational stability, efficient conversion under industrially relevant feeds, direct utilization of solar irradiation or improvements in photon‐generation efficiency, and ideally the elimination of noble metals. Although Co‐based systems have shown strong performance, cobalt is classified as a critical raw material [110], introducing possible supply risks and price volatility. Catalysts based on earth‐abundant and geopolitically more secure elements, such as Ni or Cu, therefore represent especially attractive targets. Future progress will require catalytic performance, reactor design, techno‐economic viability, and supply‐chain constraints to be integrated from the earliest stages of material development.

## 5 Summary and Outlook

Recent advances in photocatalytic acetylene semi‐hydrogenation have established this reaction as a promising nonthermal strategy for producing polymer‐grade ethylene [111]. When combined with upstream technologies for acetylene generation from non‐oil feedstocks, this approach should extend the role of acetylene beyond that of an impurity in crude ethylene streams, positioning it as a potential feedstock for ethylene production [112, 113, 114]. Although electrocatalytic systems have advanced rapidly in this context [26, 106], their practical implementation is still limited by high overpotentials, modest energy efficiency, and competing side reactions [25, 27, 115]. Photocatalytic systems, particularly those reported to operate through HAT or CPET pathways, offer an attractive complementary strategy because they can achieve high activity and selectivity under mild conditions, opening opportunities for sunlight‐driven acetylene‐to‐ethylene conversion and, ultimately, more sustainable plastic production. However, the CPET pathway has not yet been fully taken advantage of, apart from the single heterogeneous example discussed above [82], and clearly deserves further investigation. The operative mechanism is governed by a complex interplay between catalyst electronic structure and reaction environment, in which catalyst design can play a decisive role in determining the preferred mechanistic pathway. For HAT pathways, for example, a Cosalen derivative bearing electron‐donating ligands [66], as well as cobaloximes with electron‐donating substituents [67], have been shown to energetically favor cobalt–hydride formation and improve ethylene formation performance. At the same time, the overall reaction environment, including the solvent and pH, can also play a key role. For example, homogeneous Co porphyrins in aqueous solution proceed through cobalt–acetylene π‐complex intermediates [56], whereas Co porphyrins incorporated as MOF linkers and used in acetonitrile have been proposed to operate through HAT [61].

The direct utilization of solar energy could have a significant impact on the economics of photocatalytic processes [64]. Improving the overall energy efficiency of the process could strengthen one of the key advantages of photocatalysis over both thermocatalytic and electrocatalytic approaches by reducing the contribution of energy costs to the overall process economics. Despite this progress, several challenges remain before photocatalytic acetylene semi‐hydrogenation starts to be adopted in industry as an alternative to established thermocatalytic processes. Current systems often still rely on organic solvents, sacrificial electron donors that generate waste, and catalysts that degrade under extended operation. Homogeneous catalysts also present practical limitations with respect to catalyst recover, recycling, and industrial implementation. At the same time, they remain particularly valuable because their activity and selectivity can be systematically tuned through ligand modification, providing mechanistic insight that is difficult to obtain from heterogeneous materials alone. The knowledge gained from these studies has already provided, and will continue to provide, a foundation for the rational design of increasingly efficient catalytic systems, first homogenous and subsequently heterogeneous ones. Addressing the remaining limitations will therefore require new system design rather than incremental improvements alone. One promising direction is the combination of selective adsorption and photocatalytic activity within porous materials, which could increase the local acetylene concentration at the catalytic interface and enhance efficiency. Ideally, sacrificial electron donors should be eliminated altogether through system designs that achieve full redox balance, for example by integrating photocatalysis with electrochemical or photoelectrochemical platforms, or by taking advantage of the spatial proximity of oxidants and reductants in light‐driven reactions to integrate both redox processes [116].

A key opportunity is the integration of molecular and supramolecular catalysts into electrodes and photoelectrodes, bridging the mechanistic precision of homogeneous systems with the recovery, stability, and continuous‐operation advantages of heterogeneous devices. This strategy is well established in related areas such as CO_2_ reduction and H_2_O reduction, where molecular catalysts have been immobilized on conductive or semiconductive supports and incorporated into electrolyzers or photoelectrochemical cells [117, 118, 119]. Similar architectures could be highly valuable for acetylene semi‐hydrogenation: conductive supports would enable controlled charge delivery and catalyst recycling, while semiconductor photoelectrodes could use photogenerated charges to drive acetylene reduction without soluble **PS**s or **SD**s. In an ideal configuration, acetylene reduction at a cathode or photocathode would be coupled to water oxidation or, more attractively, to a value‐generating anodic oxidation reaction, improving both atom economy and process economics. Toward this goal, photocatalytic systems have already shown that acetylene reduction can be coupled to the oxidative upgrading of alcohols, amines, biomass‐derived substrates, or other organic feedstocks [58, 64]. These examples provide a conceptual basis for developing paired electrochemical or photoelectrochemical platforms, but their translation will require careful control of catalyst immobilization, electronic communication, local proton activity, gas transport, and catalyst tolerance under realistic feed conditions. Such architectures could ultimately convert the mechanistic knowledge gained from molecular and supramolecular systems into recyclable, scalable, and device‐compatible photocatalytic platforms.

Future progress will also require investigation under increasingly realistic operating conditions. In particular, systematic studies of catalyst tolerance toward gas impurities have begun to attract attention within the photocatalysis community [56, 66], but remain largely absent from the field. Yet these studies represent an important future research direction, because assessing the impact of CO and other contaminants is essential to evaluate catalyst tolerance. Moving forward, the field will also require standardized criteria for performance evaluation, including a unified ppm‐based benchmark that explicitly states whether acetylene concentrations below 5 ppm are achieved, together with product analysis that extends beyond the gas phase to include the systematic identification and quantification of possible liquid‐phase by‐products. Although photocatalytic semi‐hydrogenation of substituted alkenes and alkynes represents an important and growing research area [14, 94, 120, 121], acetylene remains a uniquely informative substrate because it combines stringent selectivity requirements with direct industrial relevance. At the same time, the mechanistic understanding gained from acetylene semi‐hydrogenation, including insights into hydrogen‐transfer pathways, substrate selectivity, and catalyst design, may help guide both the extension of photocatalytic alkyne semi‐hydrogenation to more complex substrates and the development of the complementary reverse transformation from alkenes to alkynes [122, 123]. Ultimately, both conventional ethylene purification and emerging acetylene‐based production routes require catalysts capable of complete C_2_H_2_ conversion with near‐quantitative C_2_H_4_ selectivity under energy‐efficient conditions. Achieving this goal will depend on robust, earth‐abundant catalytic materials, improved light management, continuous‐flow reactor engineering, and techno‐economic considerations integrated from the earliest stages of catalyst design.

## Author Contributions

**Anna Fortunato**: writing – original draft, writing – review and editing. **Brando Adranno**: writing – original draft, writing – review and editing. **Sharon Silloni**: writing – original draft, writing – review and editing. **Marianna Barbieri**: writing – original draft, visualization. **Claudia Masia**: writing – original draft, writing – review and editing. **Francesca Arcudi**: funding acquisition, writing – original draft, conceptualization, writing – review and editing, supervision, resources, project administration, methodology. **Luka Đorđević**: conceptualization, methodology, supervision, resources, project administration, writing – review and editing, writing – original draft, funding acquisition, visualization.

## Conflicts of Interest

The authors declare no conflicts of interest.

## Data Availability

Data sharing not applicable to this article as no datasets were generated or analyzed during the current study.
